# Plant Ecological Strategies in Relation to Environmental Factors Along Elevational Gradients on Gongga Mount

**DOI:** 10.3390/plants15162433

**Published:** 2026-08-10

**Authors:** Kanglong Zhu, Hong Li, Hua Lin, Dewei Li, Hongying Li, Yanling Peng, Hede Gong

**Affiliations:** 1 College of Soil and Water Conservation, Southwest Forestry University, 300 Bailongsi Road, Qingyun Sub-District, Panlong District Kunming, Kunming 650224, China; 17787032805@163.com; 2 Kunming Natural Resources Comprehensive Investigation Center, China Geological Survey, Kunming 650111, China; 3 Technology Innovation Center for Natural Ecosystem Carbon Sink, Ministry of Natural Resources, Kunming 650111, China; 4 Yunnan Technology Innovation Center of Natural Resources Carbon Sink Investigation and Carbon Asset Assessment, Kunming 650224, China; 5 Xishuangbanna Tropical Botanical Garden, Chinese Academy of Sciences, Menglun Town, Mengla County, Xishuangbanna 666303, China; 6 School of Geography and Tourism, Qilu Normal University, 2 Wenbo Road, Zhangqiu District, Jinan 250200, China

**Keywords:** plant CSR strategy, elevational gradient, leaf functional traits, Gongga Mountain region

## Abstract

The elevational divergence of plant CSR (Competitor–Stress-Tolerator–Ruderal) strategies represents a core theme in global change ecology, yet adaptive patterns across subalpine transition zones remain insufficiently understood. This study focused on 375 plant individuals sampled along an 1143–4361 m elevational gradient on Gongga Mountain. We divided elevations into three zones (low, mid, high) using tertile partitioning, measured leaf functional traits including leaf area (LA), specific leaf area (SLA), and leaf dry matter content (LDMC), and quantified CSR strategy scores with the StrateFy approach. We further explored how hydrothermal factors drive community- and intraspecific-level strategy variation. Results showed that CSR strategies differed significantly along the elevational gradient. The relative abundance of individuals with the C strategy was 53.4% at low elevations, peaked at 72.4% at mid elevations, and decreased to 43.6% at high elevations. By contrast, the S strategy was most abundant at high elevations (32.5%), while the R strategy consistently accounted for less than 5% across all zones. Life form-specific strategies varied strongly: herbs maintained a dominant C strategy (66.7%) at high elevations; trees shifted to S strategy dominance (60%) at high elevations; and shrubs displayed a balanced C–S strategy. Intraspecific plasticity in CSR strategies was pronounced: with increasing elevation, the dominant strategy shifted from C (33.3%) to S (66.7%), with a greater magnitude of variation than at the community level. Temperature was positively correlated with the C strategy (*p* < 0.001, R^2^ = 0.53) and negatively correlated with the R strategy (*p* < 0.001, R^2^ = 0.40). Precipitation was negatively correlated with the C strategy (*p* = 0.001, R^2^ = 0.54) and positively correlated with the R strategy (*p* = 0.005, R^2^ = 0.42), whereas both factors exerted weak effects on the S strategy. Our findings highlight that shifts in hydrothermal conditions are the primary drivers shaping CSR strategy distributions, and coordinated leaf trait variation serves as a key adaptation mechanism. These results improve mechanistic understanding of mountain plant adaptation and provide a scientific basis for alpine vegetation conservation and management.

## 1. Introduction

Under global climate change, mountain ecosystems exert profound influences on the spatial patterns of plant diversity due to their prominent vertical environmental gradients [1,2], serving as ideal natural laboratories for disentangling plant responses and adaptive mechanisms to environmental variation [3]. Ecological strategies developed by plants under long-term environmental filtering determine their geographic distribution and population persistence, representing a central topic in plant ecology [4]. In 1977, Grime first proposed the CSR ecological strategy framework, which classifies plant adaptive strategies into three primary types: Competitor (C), Stress-Tolerator (S), and Ruderal (R) [5]. C-strategy species achieve competitive dominance in resource-rich habitats via rapid resource acquisition; S-strategy species adapt to persistent environmental stress through resource-conservative traits; and R-strategy species cope with frequent disturbance through rapid regeneration. This theory provides a core framework for quantifying plant adaptive strategies and has become a classic paradigm in global plant functional ecology [3].

Building on this foundation, Pierce et al. compiled global plant trait datasets and developed StrateFy, a standardized framework to calculate CSR strategies using three core leaf functional traits: leaf area (LA), leaf dry matter content (LDMC), and specific leaf area (SLA) [6]. This tool greatly facilitated the worldwide application of CSR theory across diverse biomes. Accumulating evidence reveals that divergence in plant CSR strategies is tightly associated with environmental factors including climate, topography and soils, and can well explain the spatial distribution of plants along elevational and latitudinal gradients [7,8]. For instance, field observations along alpine elevational gradients demonstrate that increasing elevation significantly reduces the proportion of competitive (C) strategies while enhancing the dominance of stress-tolerant (S) strategies [9]. Global-scale analyses further confirm that CSR functional trade-offs constitute a core functional dimension governing the geographic distribution of vascular plants [8]. Meanwhile, coordinated variation in plant functional traits underlies CSR strategy formation, regulated jointly by hydrothermal conditions and soil nutrients [10], and high-altitude species exhibit more stable trait variation compared with low-altitude species and widely distributed species [11,12]. Studies indicate that high SLA and low LDMC correspond to fast resource acquisition typical of C strategies, whereas low SLA and high LDMC reflect resource conservation characteristic of S strategies. This pattern has been widely validated by the global leaf economics spectrum [13,14]. On the other hand, plants adopt adaptive trait modifications such as reduced leaf area and increased leaf thickness in response to high-altitude environmental changes [15]. Therefore, exploring linkages between leaf traits and environmental factors allows systematic dissection of environmental drivers of CSR strategies and deepens understanding of plant adaptation to changing environments [3].

Gongga Mountain, located in the eastern Qinghai–Tibet Plateau, is the highest peak of the Hengduan Mountains. Its eastern slope supports a complete vertical vegetation succession from subtropical evergreen broadleaved forests to alpine shrub meadows, with continuous and pronounced elevational divergence in temperature, precipitation, and other environmental factors, making it an exceptional natural platform for studying plant responses to steep environmental gradients [16]. However, existing studies on alpine ecosystems mostly focus on single gradients, local scales [17], and variations in leaf functional traits [18,19,20], leaving critical inadequacies in research concerning elevational patterns of plant CSR ecological strategies.

First, most elevational CSR studies focus on alpine meadows or single life forms, lacking comparative analyses across multiple life forms in subtropical–alpine transition zones. Second, research has largely focused on interspecific patterns, while the contribution and adaptive significance of intraspecific phenotypic plasticity to elevational CSR divergence remain poorly understood. Third, under the unique hydrothermal gradient of Gongga Mountain, the relative roles and regulatory mechanisms of temperature and precipitation in influencing CSR strategy differentiation remain unclear. Therefore, systematic clarification of elevational patterns and environmental influences of plant CSR strategies on Gongga Mountain is scientifically critical for refining the theoretical framework of mountain CSR strategies and revealing plant adaptation to vertical environmental gradients.

Accordingly, this study investigated plant communities along an elevational gradient from 1143 m to 4361 m on the eastern slope of Gongga Mountain. To meet the homogeneity of variance assumption for intergroup statistical comparisons and avoid statistical biases caused by uneven sample sizes among natural vegetation zones, the elevational gradient was divided into three groups (low, medium, and high elevation) using the tertile method. We measured three core leaf functional traits (LA, SLA, and LDMC) via field sampling and laboratory analysis, and quantified CSR strategies using the StrateFy approach. We systematically analyzed elevational patterns in plant CSR strategies and identified their environmental influences using mean annual temperature and annual precipitation data. This study aimed to address two key scientific questions: (1) What are the elevational patterns of plant CSR strategies on Gongga Mountain, and do responses differ among life forms? (2) Do environmental factors exert divergent effects on the differentiation of plant CSR ecological strategies?

## 2. Materials and Methods

### 2.1. Study Area

**Figure 1:**
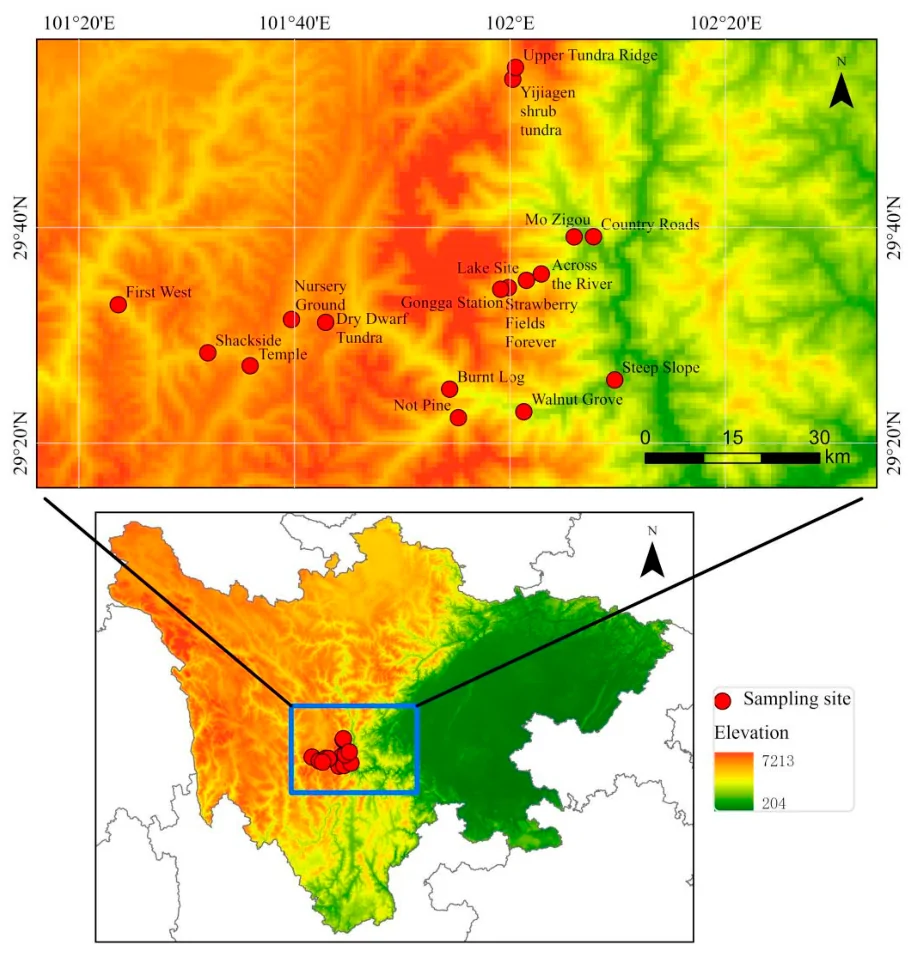
Location of the study area and distribution of sampling sites on Gongga Mountain. Note: The map shows the spatial distribution of 17 sampling sites along the 1143–4361 m elevational gradient on the eastern slope of Gongga Mountain, the upper panel shows the zoom-in visualization of the study area.

The study was conducted on the eastern slope of Gongga Mountain, Sichuan, China (101°39′42″–102°16′19″ E, 29°37′26″–30°31′45″ N), the main peak of the Hengduan Mountains at the eastern margin of the Qinghai–Tibet Plateau (Figure 1). With a maximum elevation of 7556 m a.s.l., this region forms a climatic transition zone between the humid subtropical monsoon climate of eastern China and the cold alpine climate of the Qinghai–Tibet Plateau. Climate, soil, and vegetation exhibit strong elevational zonation with continuous shifts in hydrothermal conditions, supporting a complete vertical vegetation spectrum: 1100–2200 m: subtropical evergreen broad-leaved forest zone, 2200–2500 m: montane mixed coniferous-broadleaved forest zone, 2500–3600 m: subalpine coniferous forest zone, 3600–4600 m: alpine shrub and meadow zone [21,22].

### 2.2. Methods

#### 2.2.1. Sample Collection

Field sampling was conducted during the peak growing season in July 2018 and August 2019. A total of 17 sampling sites were established between 1143–4361 m a.s.l. on the eastern slope of Gongga Mountain (five at low elevations, five at mid elevations, and seven at high elevations) to adequately represent the continuous environmental gradient. The number of sampled species per plot ranged from 13 to 30. Within each site, a stratified random sampling design was employed, and plot size was standardized according to vegetation zone to ensure representative sampling: 10 m × 10 m in low-elevation evergreen broad-leaved forests; 20 m × 20 m in mid-elevation mixed coniferous-broadleaved and subalpine coniferous forests; 5 m × 5 m in high-elevation alpine shrub meadows. Plots were placed away from obvious human disturbance, landslides, and other atypical habitats. Within each plot, mature, healthy, and undamaged individuals (free of disease, pests, or mechanical injury) were selected for leaf collection. Sun-exposed leaves from fully illuminated upper canopy positions were sampled for most species. For shade-tolerant and understory species, individuals growing in relatively well-lit conditions and isolated from conspecific competition were used. For each individual, five fully expanded, intact leaves were collected from the same canopy position, sealed in plastic bags with moist paper towels, and stored in a cool, dark box for laboratory processing within 24 h. For each species at each site, measurements were taken on three individuals with three technical replicates, and mean values were used in subsequent analyses.

In total, 375 individuals were sampled across the 17 sites, including 96 trees, 170 shrubs, and 109 herbs, representing 243 species (see Additional File S1 for the full species list).

These species make up 90.3% of the 269 newly recorded Gongga Mountain plant species contained in the second version of the China Plant Trait Database (CPTDv2). Of these, 78 species occurred and were sampled at two or more sites, enabling robust analyses of intraspecific strategy plasticity along the elevational gradient. At each site, geographic coordinates, elevation, vegetation type, and key habitat characteristics were recorded.

Moreover, the core aim of this study is to explore the overall pattern of community-scale ecological strategies across the elevational gradient of Gongga Mountain. According to Grime’s Mass Ratio Hypothesis, the aggregate functional properties of plant communities are primarily determined by dominant species [23]. Rare and associated species have little influence on community functional composition. Woody plants have large individual sizes and low species abundance within sampling plots, so we achieved adequate sampling coverage for this group. By contrast, herbaceous plants show extremely high species diversity and cannot be fully surveyed. For this reason, we adopted targeted sampling focusing on dominant herbaceous species.

#### 2.2.2. Leaf Trait Measurements

Upon returning to the laboratory, leaf samples were rehydrated for 15 min, then flattened and placed on a white background with a 1-cm scale rule. Leaves were scanned using a CanoScan 9000 F Mark II scanner (Cano Inc., Tokyo, Japan), and leaf area (LA) was determined using ImageJ software (Version 5.2). Leaf fresh weight (FW) was measured immediately with an electronic balance with a precision of 0.001 g (Shanghai Hengping Instrument Technology Co., Ltd., Shanghai, China). Leaves were then dried to constant mass in an oven at 75 °C for 48 h, after which leaf dry weight (DW) was weighed. To reduce measurement error, the mean value of five leaves per individual was used in all calculations. Specific leaf area (*SLA*, cm^2^·g^−1^) and leaf dry matter content (*LDMC*, g·g^−1^) were calculated as follows:

(1) $$\mathrm{SLA}\;=\;\frac{\mathrm{LA}\left(cm^{2}\right)}{\mathrm{DW}\left(g\right)}$$

(2) $$\mathrm{LDMC}=\frac{\mathrm{DW}\left(g\right)}{\mathrm{FW}\left(g\right)}$$

#### 2.2.3. Meteorological Data Sources

Mean annual temperature (MAT) and mean annual precipitation (MAP) were obtained from the National Ecosystem Science Data Center (https://doi.org/10.11922/sciencedb.945; https://cstr.cn/31253.11.sciencedb.945; accessed on 6 August 2025). These data were collected by the Gongga Mountain Alpine Ecosystem Observation and Experiment Station of the Chinese Academy of Sciences following the standardized automatic monitoring protocols of the Chinese Ecosystem Research Network (CERN) [24].

### 2.3. Data Processing and Statistical Analyses

#### 2.3.1. Calculation of CSR Ecological Strategies

Plant CSR strategies were quantified using the StrateFy tool developed by Pierce et al. [6]. Leaf area (LA), leaf dry matter content (LDMC), and the reciprocal of specific leaf area (1/SLA) were standardized to calculate relative CSR strategy scores (ranging from 0% to 100%). Using R software (version 4.5.1), all individuals were classified into seven strategy types (C, S, R, CS, CR, SR, and SCR) according to Grime’s CSR theory and the criteria of Pierce et al.

#### 2.3.2. Calculation of Community-Level Ecological Strategy Values

Based on CSR strategy scores, samples were grouped by sampling sites and stratified into three life forms: trees, shrubs and herbs. For widespread species that occurred at no less than two elevations within each group, the arithmetic mean values of C, S and R scores were calculated respectively. We obtained community-level values of competitive strategy (Mean-C), stress-tolerant strategy (Mean-S), and ruderal strategy (Mean-R) using the following formula:

(3) $$Mean\_Trait=\frac{1}{n}\sum_{i=1}^{n}{Strategy}_{i\;}$$

In the formula, *Mean_Trait* represents community-level strategy values (Mean-C, Mean-S, Mean-R); *n* stands for the species number within the corresponding group; and *Strategy_i_* represents the CSR strategy score of the *i*-th species. Notably, unlike abundance-weighted Community-Weighted Mean (CWM), the community arithmetic mean does not incorporate species cover or abundance weights, which may slightly weaken the dominant influence of highly dominant species on community functional attributes. Nevertheless, the species selected in this study are widespread taxa with limited differences in dominance, so the outputs show small deviations from true community CWM values and will not substantially alter the core pattern of CSR strategy divergence along the elevational gradient.

#### 2.3.3. Data Visualization

All statistical analyses and plotting were performed in R version 4.5.1. Ternary diagrams of CSR strategies were produced using the ggtern package. Linear regression plots and bar charts were constructed with the ggplot2 package. The spatial distribution of sampling sites was mapped using ArcGIS 10.7.

## 3. Results

### 3.1. CSR Strategy Distribution of Plant Communities Along Elevational Gradients on Gongga Mountain

**Figure 2:**
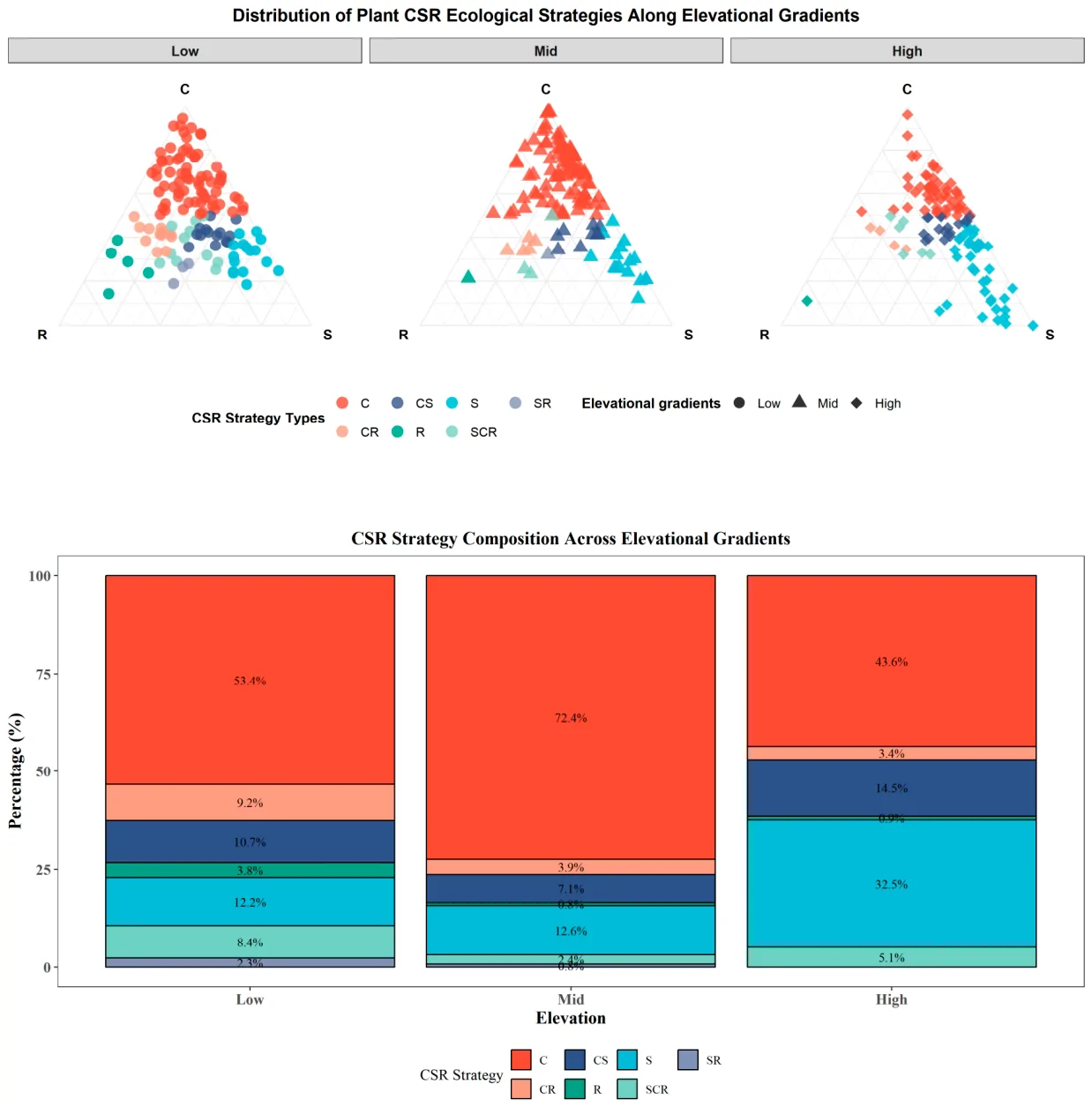
Distribution of plant individual CSR strategies across elevations. Note: Low, mid, and high elevations were defined using tertile partitioning. C: Competitor strategy; S: Stress-Tolerator strategy; R: Ruderal strategy; CS, CR, SR, and SCR: Mixed strategies. Bars represent the relative abundance (%) of individuals with each strategy.

Plant CSR strategy composition exhibited strong elevational differentiation across the 1143–4361 m gradient (Figure 2). At low elevations, communities were dominated by individuals with a competitive strategy (C, 53.4%), followed by stress-tolerant (S, 12.2%) and ruderal (R, 3.8%) strategies. Among mixed strategies, CS (10.7%) was more prevalent than CR (9.2%), SCR (8.4%), and SR (2.3%).

With increasing elevation, the relative abundance of the C strategy increased significantly and peaked at mid elevations (72.4%), then decreased sharply to 43.6% at high elevations. In contrast, the proportions of S strategy (12.6% at mid elevations → 32.5% at high elevations) and CS strategy (7.1–14.5%) showed the opposite pattern: a slight decrease at mid elevations followed by a marked increase at high elevations. The relative abundances of R strategy and mixed strategies (CR, SR, SCR) remained low and stable across the entire elevational gradient, with no significant shifts.

### 3.2. Differences in CSR Strategy Responses Among Life Forms

**Figure 3:**
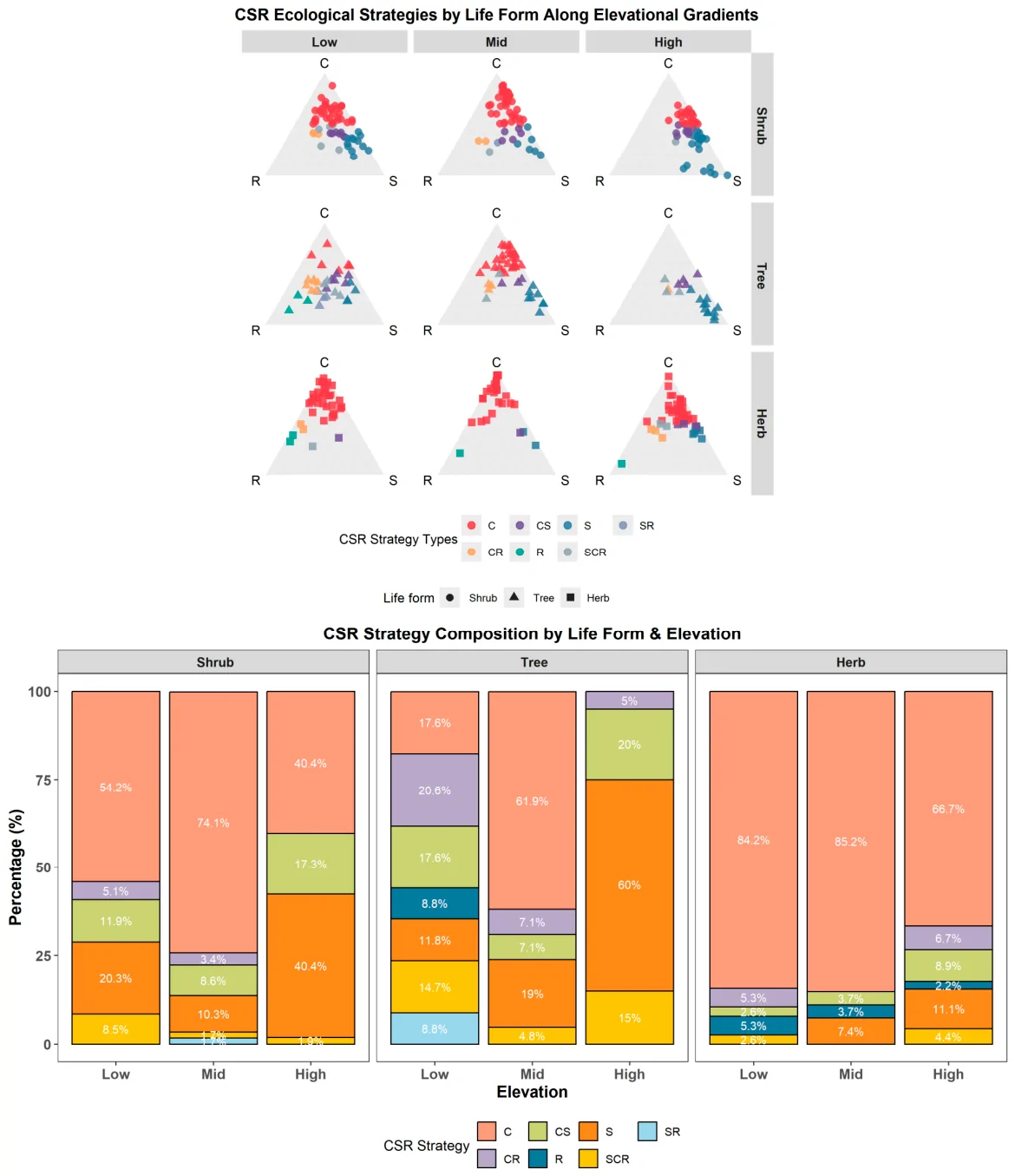
Distribution of plant individual CSR strategies among different life forms. Note: Low, mid, and high elevations were defined using tertile partitioning. C: Competitor strategy; S: Stress-Tolerator strategy; R: Ruderal strategy; CS, CR, SR, and SCR: Mixed strategies. Bars represent the relative abundance (%) of individuals with each strategy.

The three dominant life forms (trees, shrubs, and herbs) exhibited strongly life form-specific CSR strategy responses to the elevational gradient (Figure 3).

For shrubs, individuals at low elevations were dominated by the C strategy (54.2%), with the S strategy accounting for 20.3%. At mid elevations, the C strategy increased further to 74.1%, while the S strategy decreased significantly to 10.3%. At high elevations, the C strategy dropped sharply to 40.4%, and the S strategy increased correspondingly to 40.4%; meanwhile, the CS strategy rose markedly from 8.6% to 17.3%, indicating a balanced C-S strategy at high elevations.

For trees, no single strategy dominated at low elevations, where mixed strategies prevailed: CR (20.6%), C (17.6%), and CS (17.6%) showed similar proportions, followed by SCR (14.7%). At mid elevations, the C strategy became absolutely dominant (61.9%), and all other strategies were substantially reduced. At high elevations, a fundamental strategy shift occurred: the S strategy became dominant (60%), with only the CS strategy maintaining a notable proportion (20%).

For herbs, the C strategy was dominant across the entire elevational gradient, reaching 84.2% and 85.2% at low and mid elevations, respectively. Although the C strategy decreased slightly at high elevations (66.7%), it remained far higher than the S strategy (11.1%). The S strategy showed a modest but significant increasing trend with elevation (0% at low elevations → 7.4% at mid elevations → 11.1% at high elevations). No individuals exhibited the R strategy, and mixed strategies remained below 5% across all elevations.

### 3.3. Intraspecific CSR Strategy Plasticity

To further explore plant phenotypic plasticity and adaptation, 78 species distributed across different elevational gradients were selected for intraspecific CSR strategy analysis.

**Figure 4:**
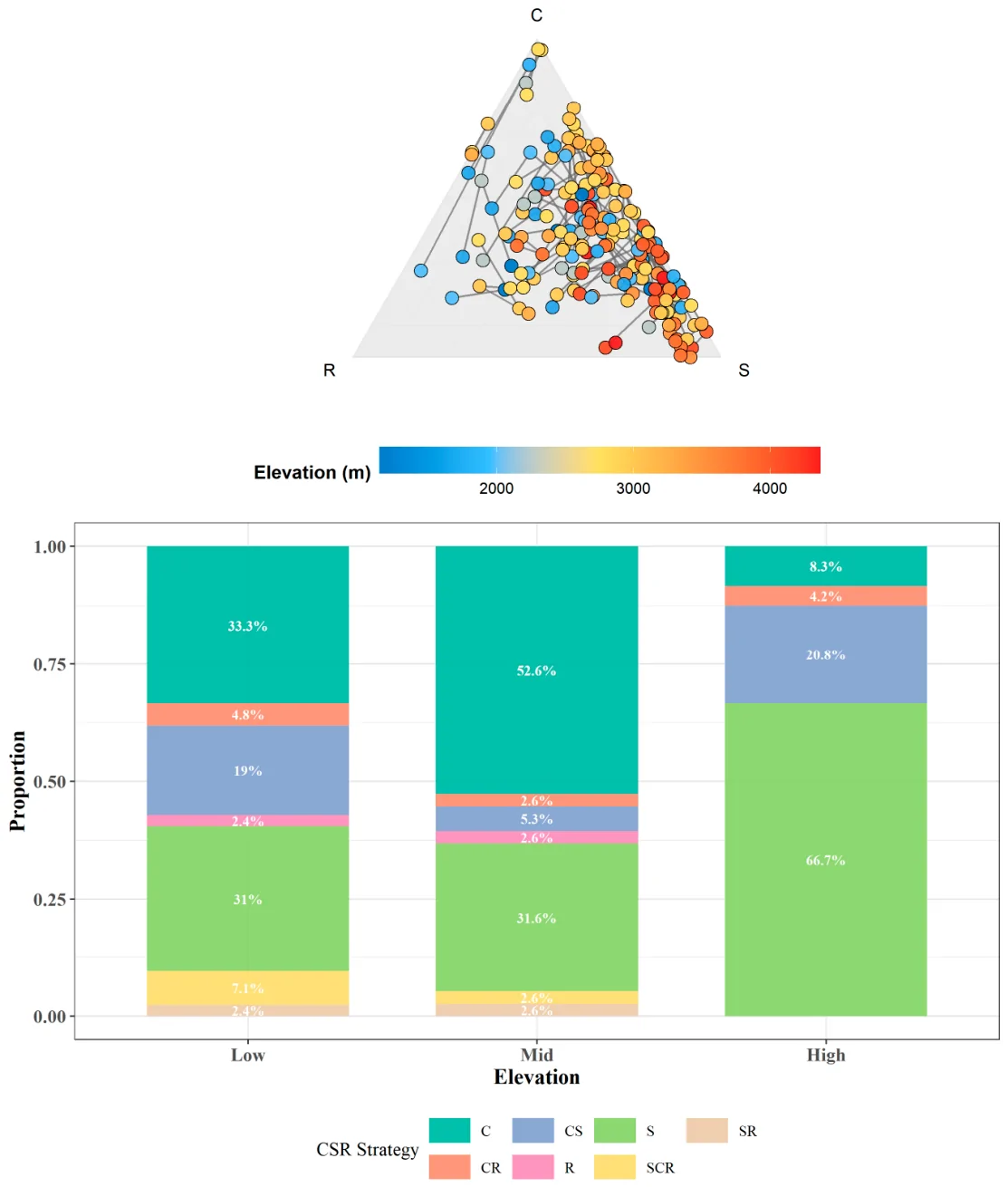
Distribution of intraspecific plant CSR strategies. Note: low, mid, and high elevations were defined using tertile partitioning. C: Competitor strategy; S: Stress-Tolerator strategy; R: Ruderal strategy; CS, CR, SR, and SCR: Mixed strategies. Circles represent individual species; circles connected by the same line illustrate the strategy shifts of a given species across elevational gradients.; bars represent the relative abundance (%) of individuals with each strategy.

Results showed that intraspecific CSR strategies exhibited strong elevational plasticity, with a significantly greater magnitude of strategy shift than that observed at the community level. At low elevations, shared species were dominated by the C strategy (33.3%) and S strategy (31.0%), followed by the CS strategy (19.0%). At mid elevations, the proportion of the C strategy increased significantly to 52.6%, the CS strategy dropped sharply to 5.3%, and the S strategy remained relatively stable (31.6%). At high elevations, a dramatic shift in strategies of shared species occurred: the S strategy became absolutely dominant (66.7%), the C strategy plummeted to 8.3%, and the CS strategy increased significantly again to 20.8%. The R strategy and other mixed strategies showed no significant expression across all elevations (Figure 4). Comparisons of intraspecific and community-scale shifts in CSR strategies (Figure 2) revealed that the magnitude of intraspecific strategy turnover (from low to high elevation, intraspecific C strategy decreased from 33.3% to 8.3%, while S strategy increased from 31% to 66.7%) was markedly larger than that at the community level (from low to high elevation, community C strategy declined from 53.4% to 43.6%, and S strategy rose from 12.2% to 32.5%). The summed absolute variation in C and S strategies within species reached 60.7%, whereas the corresponding value at the community level was only 30.1%. This pattern indicates stronger elevational plasticity at the intraspecific level.

### 3.4. Relationships Between Leaf CSR Strategies and Leaf Traits

Leaf area (LA), specific leaf area (SLA), and leaf dry matter content (LDMC) represent core functional traits of the leaf economics spectrum (LES). These three traits exhibit significant coordinated differentiation along the elevational gradient and are closely associated with the gradient shifts in plant CSR functional strategies.

**Figure 5:**
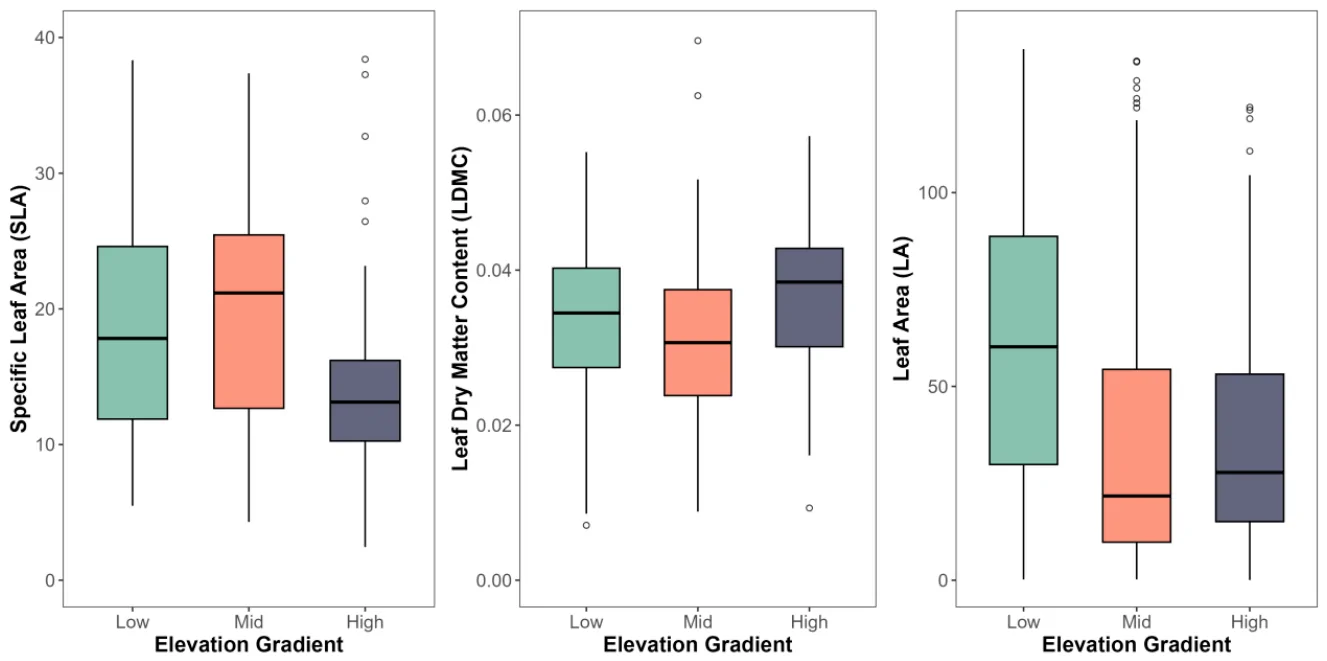
Changes in leaf functional traits along the elevational gradient. Note: Low, mid, and high elevations were defined using tertile partitioning; the black horizontal line inside each box indicates the median; the box denotes the interquartile range (25–75 percentiles), and hollow circles stand for outliers beyond this range.

Specifically, as elevation increases, SLA shows a unimodal pattern that first rises and then declines, while LDMC displays an opposite trend, decreasing initially and then increasing. By contrast, LA continuously decreases along the elevational gradient (Figure 5). Such differentiation patterns reflect divergent adaptive trade-offs between resource acquisition and environmental stress for plants inhabiting different elevational zones. Favorable hydrothermal conditions at low and mid elevations support a fast resource-acquisition trait syndrome characterized by high SLA, low LDMC and large LA. This trait combination improves light capture for photosynthesis and reduces leaf construction costs, corresponding to typical competitive (C) strategies consistent with the fast end of the LES. In contrast, low temperatures, poor soils and strong wind stress at high elevations select for conservative leaf traits including low SLA, high LDMC and small LA. These traits enhance mechanical strength, stress resistance and leaf lifespan to boost environmental tolerance, forming the physiological foundation of stress-tolerant (S) strategies aligned with the resource-conservative end of the LES.

### 3.5. Environmental Drivers of CSR Strategies

**Figure 6:**
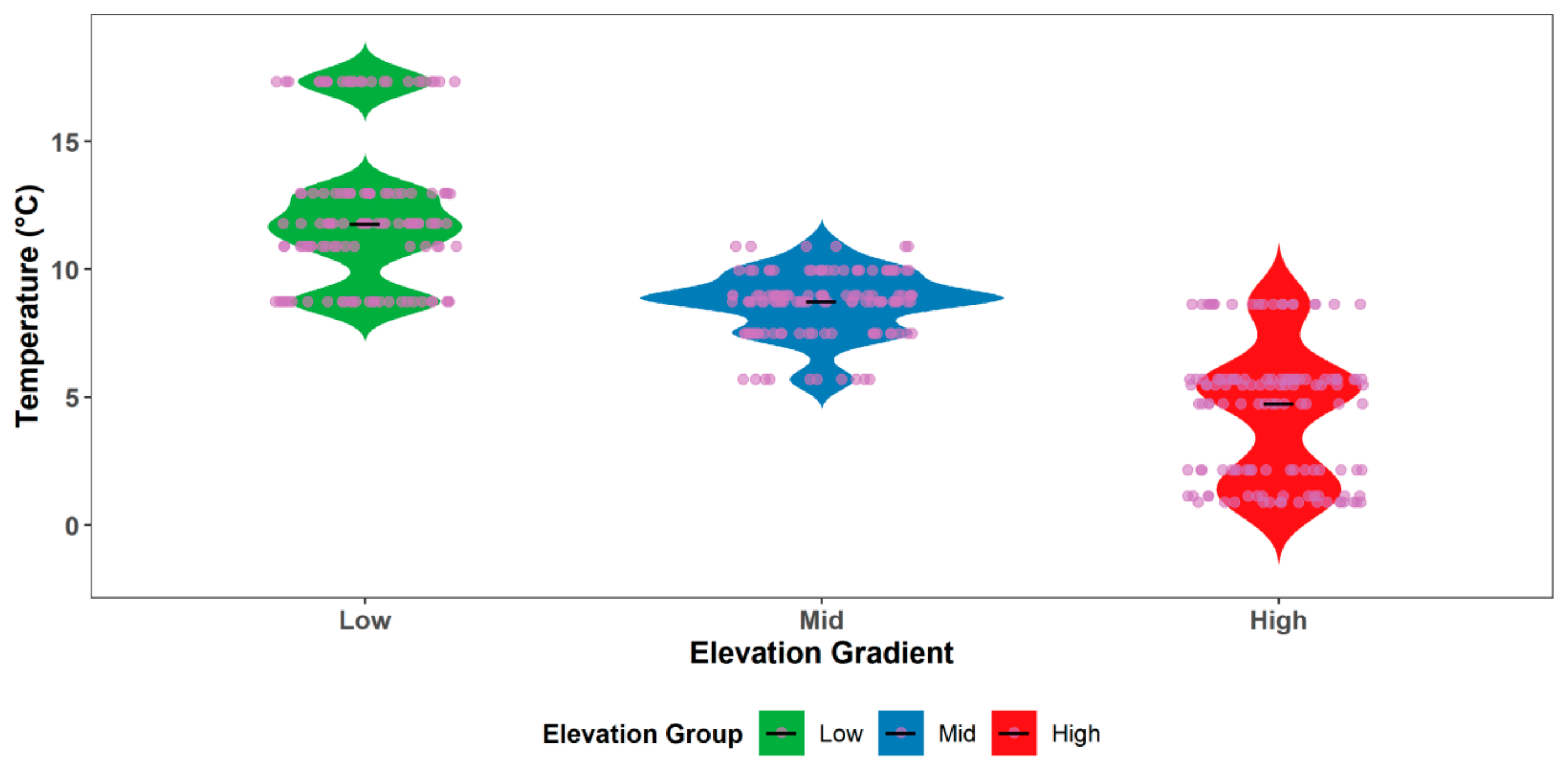
Trend of mean annual temperature (MAT) along the elevational gradient. Note: More scattered points indicate greater fluctuation; low, mid, and high elevations were defined using tertile partitioning.

**Figure 7:**
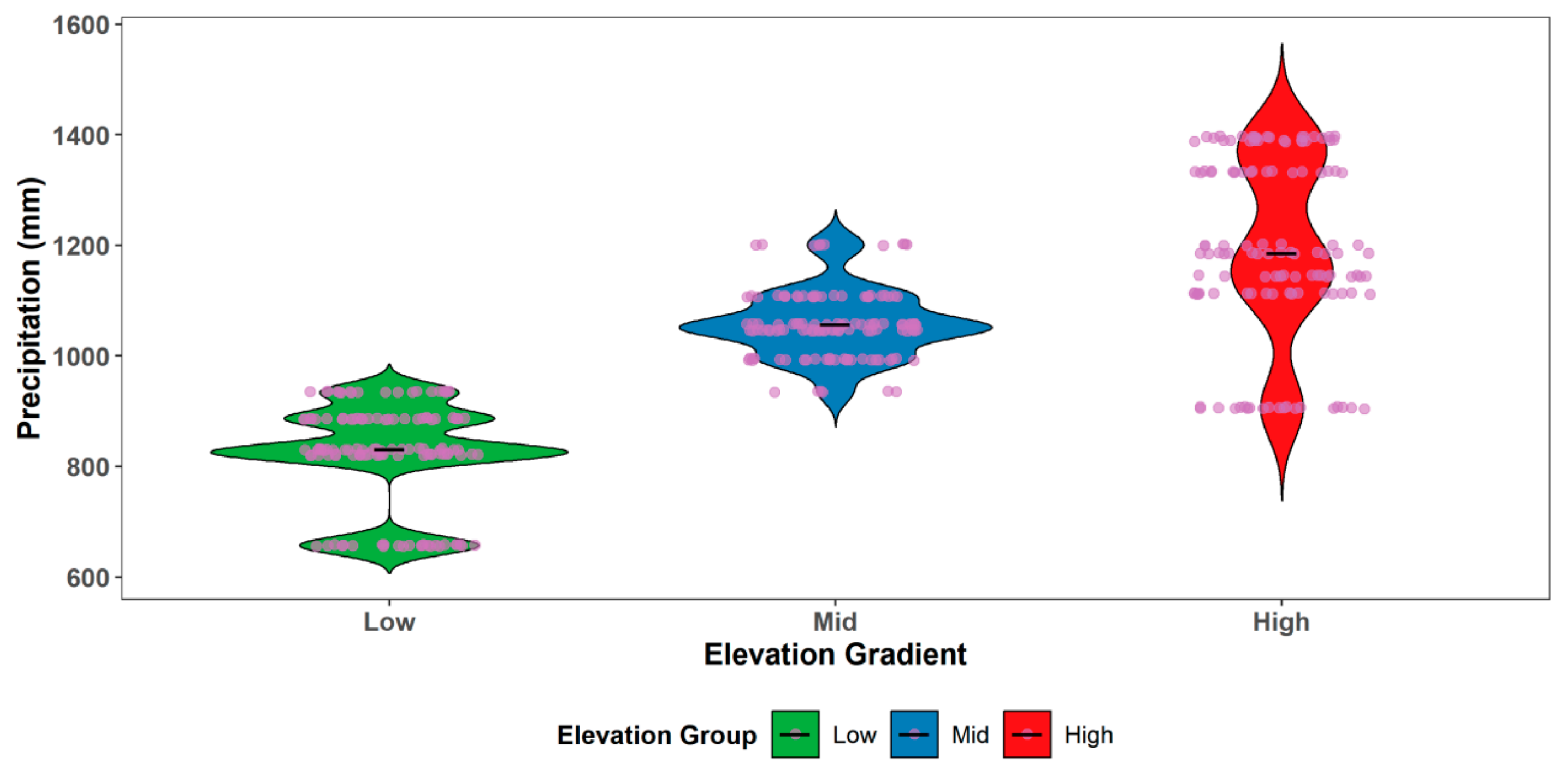
Trend of mean annual precipitation (MAP) along the elevational gradient. Note: More scattered points indicate greater fluctuation; low, mid, and high elevations were defined using tertile partitioning.

Hydrothermal conditions along the elevational gradient on Gongga Mountain changed regularly: air temperature decreased significantly with increasing elevation, while precipitation increased significantly with elevation. The mid-elevation zone exhibited the lowest spatial heterogeneity in hydrothermal conditions and the highest environmental stability (Figure 6 and Figure 7).

**Figure 8:**
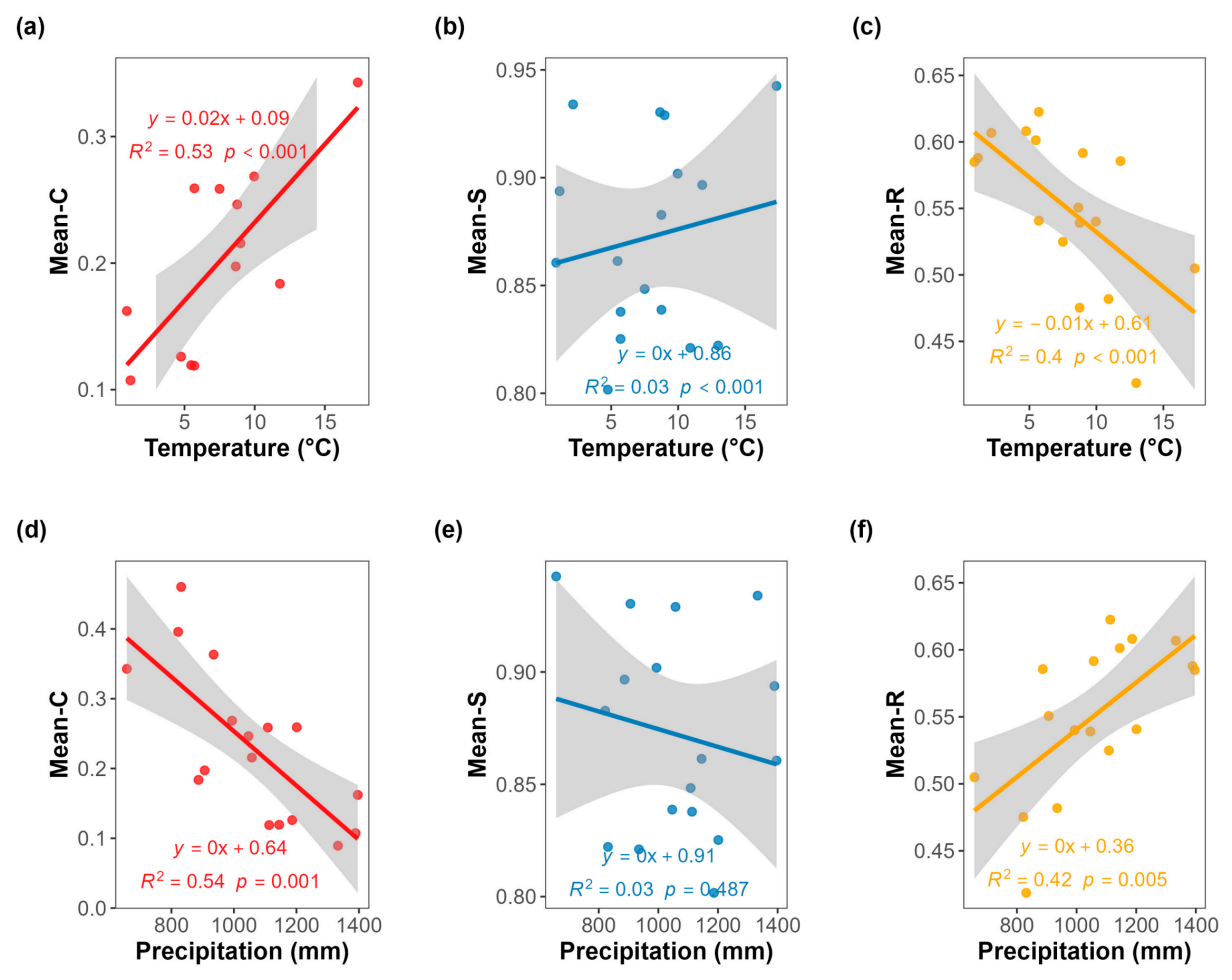
Linear regression analyses between hydrothermal factors and community-level CSR strategy values. Note: (**a**) Correlation between temperature and community − level C − strategy values; (**b**) Correlation between temperature and community − level S − strategy values; (**c**) Correlation between temperature and community − level R − strategy values; (**d**) Correlation between precipitation and community − level C − strategy values; (**e**) Correlation between precipitation and community − level S − strategy values; (**f**) Correlation between precipitation and community − level R − strategy values; R^2^ represents the proportion of variation explained by the model.

To further explore the effects of temperature and precipitation on plant community CSR strategies, linear regression models were established using arithmetic mean CSR scores at the community level. Results showed that temperature and precipitation had significant selective effects on plant community CSR strategies, exerting significant influences only on the C and R strategies, with weak explanatory power for the S strategy. Specifically, temperature was significantly positively correlated with the C strategy (R^2^ = 0.53, *p* < 0.001) and significantly negatively correlated with the R strategy (R^2^ = 0.40, *p* < 0.001). Precipitation was significantly negatively correlated with the C strategy (R^2^ = 0.54, *p* = 0.001) and significantly positively correlated with the R strategy (R^2^ = 0.42, *p* = 0.005). Linear regression models for the effects of temperature and precipitation on the S strategy were not statistically significant (R^2^ = 0.03) (Figure 8).

Overall, the warm and dry hydrothermal conditions at low elevations favored the expression of the C strategy; the stable warm and humid environment at mid elevations further strengthened the dominance of the C strategy, while the cold and humid conditions at high elevations were associated with shift in the community toward the S strategy.

## 4. Discussion

### 4.1. Spatial Distribution Characteristics of Plant CSR Strategies

CSR strategies represent adaptive responses adopted by plants to maintain growth and ensure species persistence under varying environmental conditions [5]. Specifically, C-strategy species are adapted to resource-rich environments, S-strategy species possess strong survival capabilities under resource-limited and highly stressful conditions [25], and R-strategy species thrive in disturbed habitats through rapid reproduction [26].

This study revealed significant elevational shifts in plant CSR strategies on Gongga Mountain: the relative abundance of C-strategy individuals exhibited a unimodal pattern along the elevational gradient, increasing from low to mid elevations and then decreasing at high elevations, while the S-strategy showed the opposite trend. In contrast, the R-strategy values remained below 5% at all elevations (Figure 2). This pattern is consistent with the core predictions of Grime’s CSR framework [5]. Combining the divergent patterns of leaf traits and hydrothermal conditions along the elevational gradient (Figure 5, Figure 6 and Figure 7), we infer that sufficient resources and favorable hydrothermal conditions at low elevations enable plants to gain competitive advantages via a trait syndrome of high SLA, low LDMC and large leaf area for rapid resource acquisition [13,27]. Such trait combinations accelerate nutrient uptake rates and sustain robust vegetative growth [19,28]. Meanwhile, competitive pressure for limited resources allows S strategies to occupy a certain proportion of the overall CSR spectrum. This aligns with the CSR differentiation patterns observed in subtropical forests [8], where resource-rich low-elevation habitats favor competitive strategies.

At mid elevations, stable temperature and precipitation lead to low environmental heterogeneity, which further strengthens the C strategy of plants. Severe environmental stress at high elevations, by contrast, induces shifts in plant strategies toward the S strategy that sustains stable metabolism [29,30]. This pattern holds across different plant life forms (Figure 3): C strategies of trees, shrubs and herbs all peak at mid elevations. By contrast, extreme environments characterized by low temperatures, strong winds and high precipitation at high elevations compel plants to enhance leaf structural stability and reduce resource loss to survive under nutrient limitation and intense physical stress [7,14]. Accordingly, the stress-tolerant (S) strategy dominates plant communities at high elevations in alpine zones [9].

Furthermore, from low to high elevations, the magnitude of intraspecific strategy variation across elevations reached 60.7% (C strategy decreased from 33.3% to 8.3%, and S strategy increased from 31% to 66.7%), which was greater than the 30.1% variation observed at the community level (C strategy declined from 53.4% to 43.6%, and S strategy rose from 12.2% to 32.5%). The stronger elevational plasticity of ecological strategies at the intraspecific level implies that the community-level strategy divergence may stem either from environmental filtering or intraspecific shifts within the same species. Recent studies also show that a growing number of researchers focus on patterns of intraspecific phenotypic variation along mountain elevational gradients [31,32], aiming to advance our understanding of plant strategies under steep environmental gradients.

Nevertheless, this study still presents certain limitations in terms of species coverage. Firstly, typical stress-tolerant species including *Kobresia*, *Festuca ovina* and *Stipa* spp. were not collected from high-elevation zones of Gongga Mountain. These species exert vital effects on improving the overall community stress tolerance and shaping community CSR strategy patterns under harsh high-elevation conditions. Therefore, we speculate that the absence of these species may cause biases in community strategy evaluation, and restrict the accurate reflection of the genuine ecological strategy composition of native high-elevation communities in this region. In addition, the conclusion that competitive strategy predominates among high-elevation herbs is likely influenced by the insufficient sampling of small-leaved stress-resistant species. The limited sampling coverage interferes with the objective judgment of strategy distribution characteristics of alpine herbaceous plants.

Therefore, future research should target representative stress-tolerant species and expand sampling coverage to further improve the comprehensiveness and accuracy of findings, enabling more precise identification of how alpine plant ecological strategies evolve along elevational gradients.

### 4.2. Elevational Responses of CSR Strategies Among Different Life Forms

Mount Gongga, as a typical subtropical–alpine transition zone, exhibits distinct life form-specific differences in CSR strategy responses to elevational gradients, which fills the gap in previous studies that focused on single life forms. Specifically, with increasing elevation, shrubs displayed a balanced distribution of C and S strategies at high elevations, trees shifted toward dominant stress-tolerant (S) strategies at high elevations, and herbs maintained dominant competitive (C) strategies across the entire elevational gradient (Figure 3).

This seemingly counterintuitive pattern may be closely associated with the life history traits of herbs. We speculate that herbs possess short life cycles and rapid reproductive rates [33], enabling them to occupy high-elevation microhabitats such as valleys and slope toes that buffer extreme environmental stress. These microhabitats offer relatively favorable resource conditions, allowing herbs to maintain competitive (C) strategies oriented toward rapid resource acquisition even at high elevations. In contrast, trees are long-lived woody plants exposed to persistent environmental stress at high elevations. Accordingly, they adopt stress-tolerant (S) strategies to improve structural stability and conserve resources for long-term survival [6]. Shrubs exhibit life history traits intermediate between trees and herbs and display balanced C–S strategies at high elevations, reflecting their intermediate adaptive capacity under moderate stress.

This divergent response among life forms highlights the necessity of considering species life history traits when analyzing elevational patterns of CSR strategies. Most previous elevational CSR studies have focused on single life forms, which may underestimate the complexity of community strategy composition. Although this study included multiple life forms (trees, shrubs, and herbs), liana species were not sampled. Future research should incorporate lianas to further improve the completeness of life form coverage and clarify the generality of CSR strategy patterns in subtropical–alpine transition zones.

### 4.3. Mechanisms of Environmental Factors and Species on CSR Strategies

Linear regression analyses revealed significant selective effects of temperature and precipitation on CSR strategies, consistent with the “environment-determined strategy” paradigm [34]. Specifically, temperature was positively correlated with the C-strategy (R^2^ = 0.53, *p* < 0.001) and negatively correlated with the R-strategy (R^2^ = 0.40, *p* < 0.001), indicating that warm conditions favor resource acquisition and competition, while low temperatures inhibit rapid resource utilization. Precipitation was negatively correlated with the C-strategy (R^2^ = 0.54, *p* = 0.001) and positively correlated with the R-strategy (R^2^ = 0.42, *p* = 0.005), suggesting that high precipitation may increase environmental disturbance or resource leaching, favoring ruderal strategies.

Notably, both temperature and precipitation exhibited very weak explanatory power for the S strategy (R^2^ = 0.03), suggesting that the S strategy is not primarily regulated by hydrothermal conditions alone. Instead, it is more likely shaped by a combination of comprehensive stress factors, including strong wind, intense ultraviolet radiation, and soil nutrient limitation [35,36]. Existing studies have indicated that ecosystem nutrient dynamics are modulated by soil stoichiometry [37]. At high elevations, low temperatures inhibit soil nitrogen mineralization and reduce the availability of nutrients such as nitrogen, compelling plants to adopt resource-conservative S strategies [14]. Meanwhile, strong winds and intense ultraviolet radiation damage leaf structures and facilitate the formation of stress-resistant traits (e.g., high LDMC and low SLA), further strengthening the dominance of the S strategy [38].

These findings imply that elevational differentiation of plant CSR strategies on Mount Gongga is jointly associated with direct hydrothermal effects and indirect effects arising from comprehensive stress. Temperature and precipitation directly modulate C and R strategies by altering resource availability, whereas comprehensive stressors (wind, ultraviolet radiation, and soil nutrients) are linked to spatial variation in the S strategy. This multi-factor influencing mechanism aligns with global CSR patterns, whereby multiple environmental filters jointly shape plant adaptive strategies [6]. Nevertheless, this study only verified the correlative relationships between hydrothermal conditions and plant CSR strategies, and the synergistic regulatory mechanisms of multiple factors remain unclear. Therefore, future research should further incorporate co-varying abiotic factors along elevational gradients such as soil properties and ultraviolet radiation to construct a more comprehensive dataset of environmental factors, enabling more precise disentangling of the mechanisms shaping CSR strategies of alpine plant communities. Meanwhile, we should recognize that the interactions between vegetation and environments are not unidirectional responses; instead, bidirectional feedbacks exist between them [39,40]. A unidirectional hydrothermal regulation framework alone cannot fully characterize the differentiation processes of plant strategies along mountain elevational gradients.

## 5. Conclusions

This study systematically disentangles the differentiation characteristics of plant CSR ecological strategies along the 1143–4361 m elevational gradient on Gongga Mount and explores their correlational patterns with hydrothermal factors. The core conclusions are as follows: (1) Community-level CSR strategies on Gongga Mount exhibited significant differentiation along the elevational gradient. The proportion of competitive (C) strategy followed a unimodal pattern and peaked at mid elevations, whereas the stress-tolerant (S) strategy displayed the opposite unimodal valley trend. The ruderal (R) strategy maintained an extremely low proportion across all elevations. Distinct life form-specific differences were detected in elevational responses: herbs sustained dominant competitive (C) strategies across the entire elevational gradient, trees shifted toward stress-tolerant (S) strategy dominance at high elevations, and shrubs showed balanced distributions of C and S strategies at high elevations. (2) Intraspecific CSR strategies of the selected species possessed prominent elevational plasticity. As elevation increased, the dominant strategy shifted from C to S, and the magnitude of intraspecific strategy turnover was larger than that at the community scale. This intraspecific adjustment constitutes an important pathway for plant adaptation to elevational gradients. (3) Hydrothermal factors exerted divergent influences on CSR strategies: temperature was significantly positively correlated with the C strategy, while precipitation showed a significant negative correlation with the C strategy. Temperature and precipitation exerted opposite correlative effects on the ruderal (R) strategy, and both variables exhibited weak explanatory power for the S strategy. Based on correlation analyses, elevational shifts in hydrothermal conditions represent important abiotic correlates associated with regional CSR strategy patterns.

This study offers insights for multi-scale analyses of plant CSR strategies within subtropical–alpine transition zones and supports predictions regarding mountain plant responses to climate change. Nevertheless, constrained by sampling coverage and limited environmental measurements, potential stress factors including soil nutrients, wind speed, and ultraviolet radiation were not incorporated into our models. In addition, this correlational analysis cannot strictly distinguish causal relationships. Further research integrating multi-factor monitoring is required to disentangle the mechanisms underlying CSR strategy differentiation.

## Data Availability

The raw data generated in this study (including plant functional trait measurements and field survey records) are available from the corresponding authors (Hong Li or Hede Gong) upon reasonable request.

## Supplementary Materials

The following supporting information can be downloaded at: https://www.mdpi.com/article/10.3390/plants15162433/s1.
