# Supplementary material for: Plant Ecological Strategies in Relation to Environmental Factors Along Elevational Gradients on Gongga Mount
Source: Plants (Basel). 2026 Aug 10;15(16):2433. doi: 10.3390/plants15162433 (PMC13516118; doi:10.3390/plants15162433)
Supplement: Supplementary file 1 [file plants-15-02433-s001.zip › plants-4455999-supplementary.pdf]

Additional File S1: A total of 375 plant specimens (belonging to 243 species) collected in this study are currently deposited in Southwest Forestry University. All specimens were identified by Professor Hede Gong and are assigned vouc her numbers ranging from GGS201807 to GGS201908.（All species scientific n ames were uniformly verified and standardized based on the online edition of *Flora of China*.）

Species	Lifestyle	Elevation	CSR type
Euptelea pleiosperma	Tree	1143	CR
Betula utilis	Tree	1143	CS
Cunninghamia lanceolata	Tree	1143	S
Juglans cathayensis	Tree	1143	CR
Cyclobalanopsis glaucoides	Tree	1143	S
Pyracantha fortuneana	Shrub	1143	S
Viburnum foetidum var. ceanothoides	Shrub	1143	C
Rhamnus dumetorum	Shrub	1143	S
Litsea cubeba	Shrub	1143	C
Quercus gilliana	Tree	1143	SCR
Pistacia weinmannifolia	Tree	1143	S
Trachycarpus fortunei	Tree	1143	R
Ilex corallina	Shrub	1143	S
Hydrangea xanthoneura	Shrub	1143	CR
Debregeasia orientalis	Shrub	1143	C
Rubus macilentus	Shrub	1143	CS
Myrsine semiserrata	Shrub	1143	CS
Grewia biloba	Shrub	1143	SCR
Boehmeria clidemioides var. diffusa	Herb	1143	C
Elatostema cuspidatum	Herb	1143	C
Arisaema erubescens	Herb	1143	CR
Begonia henryi	Herb	1143	C
Boenninghausenia albiflora	Herb	1143	C
Lophatherum gracile	Herb	1143	C
Woodwardia unigemmata	Herb	1143	SCR
Alnus cremastogyne	Tree	1650	CR
Cyclobalanopsis glaucoides	Tree	1650	S
Quercus serrata var. brevipetiolata	Tree	1650	SCR
Ligustrum lucidum	Shrub	1650	SCR

Rhus chinensis	Tree	1650	SR
Toxicodendron sylvestre	Tree	1650	CS
Rhamnus tangutica	Shrub	1650	C
Morus australis	Tree	1650	SR
Rhododendron augustinii	Shrub	1650	S
Lyonia ovalifolia var. lanceolata	Shrub	1650	SCR
Alangium chinense	Tree	1650	CR
Pyracantha fortuneana	Shrub	1650	CS
Debregeasia orientalis	Shrub	1650	C
Neillia affinis	Shrub	1650	C
Lespedeza formosa	Shrub	1650	C
Maclura tricuspidata	Shrub	1650	CR
Rosa glomerata	Shrub	1650	S
Agrimonia pilosa	Herb	1650	C
Arisaema erubescens	Herb	1650	C
Zingiber striolatum	Herb	1650	CR
Artemisia argyi	Herb	1650	C
Anaphalis bicolor	Herb	1650	C
Lophatherum gracile	Herb	1650	C
Pseudocyclosorus esquirolii	Herb	1650	C
Alnus ferdinandi-coburgii	Tree	1785	C
Litsea cubeba	Shrub	1785	C
Betula utilis	Tree	1785	CS
Tetracentron sinense	Tree	1785	C
Salix wallichiana	Tree	1785	C
Rhus chinensis	Tree	1785	CS
Viburnum oliganthum	Shrub	1785	S
Coriaria nepalensis	Shrub	1785	S
Indigofera szechuensis	Shrub	1785	C
Elaeagnus umbellata	Shrub	1785	C
Rubus mesogaeus	Shrub	1785	C
Debregeasia orientalis	Shrub	1785	C
Cotoneaster dielsianus	Shrub	1785	S
Aster albescens	Herb	1785	C
Pyracantha fortuneana	Shrub	1785	S
Lonicera ligustrina	Shrub	1785	C
Viburnum atrocyaneum	Shrub	1785	S
Salix variegata	Tree	1785	C
Viburnum rhytidophyllum	Shrub	1785	S
Hydrangea strigosa	Shrub	1785	C
Rubus setchuenensis	Shrub	1785	C
Rubus lambertianus	Shrub	1785	C
Galium dahuricum var. lasiocarpum	Herb	1785	C

Ficus tikoua	Herb	1785	C
Arisaema erubescens	Herb	1785	C
Begonia grandis	Herb	1785	C
Pilea pumila	Herb	1785	C
Artemisia tangutica	Herb	1785	C
Incarvillea arguta	Herb	1785	C
Alnus cremastogyne	Tree	1976	R
Juglans cathayensis	Tree	1976	R
Euptelea pleiosperma	Tree	1976	SCR
Machilus viridis	Tree	1976	SCR
Toxicodendron vernicifluum	Tree	1976	CR
Elaeagnus bockii	Shrub	1976	CS
Salix wallichiana	Tree	1976	CS
Styrax roseus	Shrub	1976	C
Sorbaria arborea	Shrub	1976	SCR
Litsea cubeba	Shrub	1976	C
Viburnum betulifolium	Shrub	1976	SCR
Rubus lambertianus	Shrub	1976	C
Rhamnus dumetorum	Shrub	1976	C
Hydrangea anomala	Shrub	1976	C
Rubus setchuenensis	Shrub	1976	CR
Neillia affinis	Shrub	1976	C
Cotoneaster dielsianus	Shrub	1976	C
Viburnum foetidum var. rectangulatum	Shrub	1976	C
Iris confusa	Herb	1976	R
Zingiber striolatum	Herb	1976	C
Agrimonia pilosa	Herb	1976	C
Artemisia argyi	Herb	1976	C
Boehmeria clidemioides var. diffusa	Herb	1976	C
Oplismenus undulatifolius	Herb	1976	C
Carex henryi	Herb	1976	C
Dryopteris neorosthornii	Herb	1976	C
Bambusa multiplex	Shrub	1976	C
Arisaema erubescens	Herb	1976	R
Lithocarpus cleistocarpus	Tree	2258	SR
Malus prattii	Tree	2258	CR
Euptelea pleiosperma	Tree	2258	C
Sorbus meliosmifolia	Tree	2258	CS
Machilus viridis	Tree	2258	SCR
Tetracentron sinense	Tree	2258	CR
Corylus ferox	Shrub	2258	C
Rhododendron polylepis	Shrub	2258	CS
Ilex pernyi	Shrub	2258	S

Ilex yunnanensis	Shrub	2258	CS
Cornus controversa	Tree	2258	C
Ilex fragilis f. kingii	Shrub	2258	C
Polygala fallax	Herb	2258	C
Rubus pentagonus	Shrub	2258	C
Ribes longiracemosum	Shrub	2258	C
Viburnum oliganthum	Shrub	2258	CS
Viburnum kansuense	Shrub	2258	C
Adenocaulon himalaicum	Herb	2258	C
Pternopetalum davidii	Herb	2258	C
Oxalis griffithii	Herb	2258	C
Rubus fockeanus	Shrub	2258	C
Calanthe tricarinata	Herb	2258	C
Carex henryi	Herb	2258	C
Polystichum braunii	Herb	2258	CS
Fargesia ferax	Shrub	2258	C
Sorbus pallescens	Tree	2735	CR
Tilia chinensis var. intonsa	Tree	2735	C
Padus buergeriana	Tree	2735	CR
Acer laxiflorum	Tree	2735	C
Rhododendron Polylepis	Shrub	2735	CS
Abies fabri	Tree	2735	C
Betula utilis	Tree	2735	CS
Larix potaninii var. marcrocarpa	Tree	2735	CS
Picea likiangensis	Tree	2735	S
Ilex pernyi	Shrub	2735	S
Lonicera lanceolata	Shrub	2735	C
Malus yunnanensis	Tree	2735	C
Viburnum betulifolium	Shrub	2735	C
Rubus pungens var. oldhamii	Shrub	2735	C
Ribes glaciale	Shrub	2735	C
Meliosma cuneifolia	Tree	2735	C
Berberis potaninii	Shrub	2735	C
Sabia yunnanensis subsp. Latifolia	Shrub	2735	C
Artemisia argyi	Herb	2735	C
Thalictrum javanicum	Herb	2735	C
Ophiopogon bodinieri	Herb	2735	S
Rubia schumanniana	Herb	2735	C
Valeriana officinalis	Herb	2735	C
Brachypodium sylvaticum	Herb	2735	S
Lepisorus thunbergianus	Herb	2735	C
Arundinaria faberi	Shrub	2735	S
Picea likiangensis	Tree	2782	S

Abies fabri	Tree	2782	C
Acer flabellatum	Tree	2782	C
Acer oliverianum	Tree	2782	C
Acer laxiflorum	Tree	2782	C
Betula utilis	Tree	2782	C
Maddenia wilsonii	Shrub	2782	C
Ilex macrocarpa	Tree	2782	C
Rhododendron calophytum	Shrub	2782	CR
Rhododendron polylepis	Shrub	2782	CS
Tsuga chinensis	Tree	2782	S
Enkianthus chinensis	Shrub	2782	C
Viburnum nervosum	Shrub	2782	C
Viburnum cylindricum	Shrub	2782	C
Malus prattii	Tree	2782	C
Euonymus semenovii	Shrub	2782	C
Litsea chunii	Tree	2782	C
Sorbus rufopilosa	Tree	2782	C
Rubus delavayi	Shrub	2782	C
Ribes glaciale	Shrub	2782	C
Cerasus trichostoma	Shrub	2782	C
Ilex yunnanensis	Shrub	2782	C
Arundinaria faberi	Shrub	2782	C
Hydrangea robusta	Herb	2782	C
Rubia cordifolia	Herb	2782	C
Pilea martini	Herb	2782	C
Oxalis griffithii	Herb	2782	C
Ligularia dentata	Herb	2782	C
Arisaema elephas	Herb	2782	C
Beesia calthifolia	Herb	2782	C
Acer laxiflorum	Tree	2950	C
Tsuga chinensis	Tree	2950	S
Betula utilis	Tree	2950	CR
Abies fabri	Tree	2950	S
Rhododendron pachytrichum	Shrub	2950	SR
Sorbus rufopilosa	Tree	2950	C
Larix potaninii var. marcrocarpa	Tree	2950	S
Cerasus trichostoma	Shrub	2950	C
Litsea chunii	Tree	2950	C
Rhododendron decorum	Shrub	2950	CS
Viburnum betulifolium	Shrub	2950	C
Cotoneaster bullatus	Shrub	2950	S
Dipelta yunnanensis	Shrub	2950	C
Sorbus pallescens	Tree	2950	SCR

Tilia chinensis var. intonsa	Tree	2950	SCR
Rubus fockeanus	Shrub	2950	C
Lonicera tangutica	Shrub	2950	CS
Rosa moyesii	Shrub	2950	C
Salix cathayana	Tree	2950	CS
Berberis potaninii	Shrub	2950	S
Buddleja davidii	Shrub	2950	CS
Parasenecio palmatisectus	Herb	2950	C
Artemisia argyi	Herb	2950	C
Gentiana robusta	Herb	2950	R
Athyrium niponicum	Herb	2950	CS
Arundinaria faberi	Shrub	2950	S
Abies fabri	Tree	2993	S
Betula utilis	Tree	2993	C
Acer kungshanense	Tree	2993	C
Acer laxiflorum	Tree	2993	C
Maddenia wilsonii	Tree	2993	C
Sorbus prattii	Tree	2993	C
Rhododendron decorum	Shrub	2993	C
Viburnum betulifolium	Shrub	2993	C
Viburnum nervosum	Shrub	2993	C
Philadelphus purpurascens	Shrub	2993	C
Cotoneaster bullatus	Shrub	2993	C
Rubus pungens var. oldhamii	Shrub	2993	C
Cerasus trichostoma	Shrub	2993	C
Euonymus frigidus	Shrub	2993	C
Padus buergeriana	Tree	2993	C
Ribes longiracemosum	Shrub	2993	C
Lonicera tangutica	Shrub	2993	C
Arundinaria faberi	Shrub	2993	C
Ribes glaciale	Shrub	2993	C
Berberis aemulans	Shrub	2993	S
Streptopus obtusatus	Herb	2993	C
Rhododendron calophytum	Shrub	2993	CR
Rosa omeiensis	Shrub	2993	C
Salvia smithii	Herb	2993	C
Arisaema elephas	Herb	2993	C
Berneuxia thibetica	Herb	2993	C
Galium hoffmeisteri	Herb	2993	C
Abies fabri	Tree	3251	S
Rhododendron calophytum	Shrub	3251	SCR
Rhododendron lutescens	Shrub	3251	C
Sorbus rufopilosa	Tree	3251	C

Acer flabellatum	Tree	3251	C
Rubus pungens	Shrub	3251	C
Lonicera tangutica	Shrub	3251	C
Lonicera nigra	Shrub	3251	C
Euonymus semenovii	Shrub	3251	C
Rosa omeiensis	Shrub	3251	C
Philadelphus purpurascens	Shrub	3251	C
Rhododendron pachytrichum	Shrub	3251	C
Maianthemum henryi	Herb	3251	C
Arisaema elephas	Herb	3251	C
Ribes glaciale	Shrub	3251	C
Acer stachyophyllum	Tree	3251	C
Carex capilliformis	Herb	3251	C
Parasenecio deltophyllus	Herb	3251	C
Acer pictum subsp. mono	Tree	3290	CR
Malus rockii	Tree	3290	SCR
Populus davidiana	Tree	3290	SCR
Cerasus pleiocerasus	Shrub	3290	CS
Picea likiangensis var. hirtella	Tree	3290	S
Sorbus thibetica	Tree	3290	SCR
Salix wallichiana	Tree	3290	CS
Rosa soulieana	Shrub	3290	S
Zanthoxylum undulatifolium	Tree	3290	CS
Rhamnus maximovicziana	Shrub	3290	C
Ribes alpestre	Shrub	3290	C
Cotoneaster tenuipes	Shrub	3290	S
Berchemia yunnanensis	Shrub	3290	CS
Quercus guyavifolia	Tree	3290	S
Chrysanthemum glabriusculum	Herb	3290	CS
Thalictrum atriplex	Herb	3290	C
Anemone tomentosa	Herb	3290	C
Salvia brevilabra	Herb	3290	C
Elsholtzia ciliata	Herb	3290	C
Onychium contiguum	Herb	3290	C
Hippophae rhamnoides	Shrub	3500	CS
Cerasus serrula	Shrub	3500	CS
Quercus guyavifolia	Tree	3500	S
Picea brachytyla	Tree	3500	S
Juniperus pingii	Shrub	3500	S
Malus transitoria	Tree	3500	S
Caragana franchetiana	Shrub	3500	S
Berberis approximata	Shrub	3500	S
Juniperus pingii var. wilsonii	Shrub	3500	S

Ribes alpestre	Shrub	3500	C
Spiraea schneideriana	Shrub	3500	C
Rhododendron intricatum	Shrub	3500	S
Halenia elliptica	Herb	3500	C
Stellera chamaejasme	Herb	3500	C
Thalictrum cultratum	Herb	3500	C
Polygonatum cirrhifolium	Herb	3500	C
Salvia prattii	Herb	3500	CR
Carex cardiolepis	Herb	3500	C
Larix potaninii var. marcrocarpa	Tree	3780	S
Picea likiangensis var. hirtella	Tree	3780	S
Pinus yunnanensis	Tree	3780	S
Hippophae rhamnoides	Shrub	3780	C
Quercus guyavifolia	Tree	3780	S
Rhododendron intricatum	Shrub	3780	C
Potentilla fruticosa	Shrub	3780	C
Juniperus pingii var. wilsonii	Shrub	3780	S
Berberis approximata	Shrub	3780	C
Caragana erinacea	Shrub	3780	S
Stellera chamaejasme	Herb	3780	C
Spenceria ramalana	Herb	3780	S
Anaphalis flavescens	Herb	3780	C
Potentilla saundersiana	Herb	3780	C
Anemone tomentosa	Herb	3780	SCR
Deyeuxia scabrescens	Herb	3780	C
Juniperus pingii var. wilsonii	Shrub	3794	S
Berberis dictyoneura	Shrub	3794	CS
Sibiraea angustata	Shrub	3794	CS
Quercus guyavifolia	Tree	3794	S
Salix wuxuhaiensis	Shrub	3794	CS
Rhododendron intricatum	Shrub	3794	S
Caragana erinacea	Shrub	3794	S
Lonicera rupicola	Shrub	3794	C
Potentilla fruticosa var. arbuscula	Shrub	3794	C
Sibbaldia cuneata	Herb	3794	S
Potentilla anserina	Herb	3794	C
Stellera chamaejasme	Herb	3794	C
Anaphalis aureopunctata	Herb	3794	C
Anemone tomentosa	Herb	3794	SCR
Carex cardiolepis	Herb	3794	C
Rhododendron phaeochrysum	Shrub	3943	S
Rhododendron intricatum	Shrub	3943	S
Berberis dictyophylla	Shrub	3943	C

Salix sclerophylla	Shrub	3943	C
Salix spodiophylla	Shrub	3943	C
Lonicera ligustrina	Shrub	3943	C
Lonicera rupicola var. syringantha	Shrub	3943	C
Spiraea schneideriana	Shrub	3943	C
Potentilla fruticosa	Shrub	3943	C
Sorbus rehderiana	Tree	3943	CS
Ribes takare	Shrub	3943	C
Picea likiangensis	Tree	3943	S
Juniperus pingii var. wilsonii	Shrub	3943	S
Rheum nobile	Herb	3943	CR
Gentiana trichotoma	Herb	3943	C
Polygonum macrophyllum	Herb	3943	C
Sedum chauveaudii	Herb	3943	C
Ligularia duciformis	Herb	3943	R
Angelica sinensis	Herb	3943	C
Rhododendron intricatum	Shrub	4081	S
Rhododendron phaeochrysum	Shrub	4081	S
Sorbus rehderiana	Tree	4081	CS
Salix sclerophylla	Shrub	4081	SCR
Potentilla fruticosa	Shrub	4081	S
Ribes takare	Shrub	4081	CS
Spiraea schneideriana	Shrub	4081	C
Lonicera ligustrina	Shrub	4081	C
Picea likiangensis	Tree	4081	S
Anaphalis souliei	Herb	4081	S
Ligularia pleurocaulis	Herb	4081	CR
Polygonum macrophyllum	Herb	4081	C
Gentiana trichotoma	Herb	4081	C
Pyrethrum tatsienense	Herb	4081	C
Potentilla stenophylla var. emergens	Herb	4081	C
Carex cardiolepis	Herb	4081	S
Rhododendron intricatum	Shrub	4361	S
Rhododendron telmateium	Shrub	4361	S
Salix flabellaris	Shrub	4361	C
Lonicera hispida	Shrub	4361	CS
Rhododendron phaeochrysum	Shrub	4361	S
Polygonum macrophyllum	Herb	4361	CS
Saussurea przewalskii	Herb	4361	C
Hedysarum vicioides	Herb	4361	C
Gentiana trichotoma	Herb	4361	C
Potentilla stenophylla var. emergens	Herb	4361	C
Trisetum spicatum	Herb	4361	CS

Carex cardiolepis	Herb	4361	S
Polystichum gongboense	Herb	4361	CS
